# Progress in Liver Transplant Tolerance and Tolerance-Inducing Cellular Therapies

**DOI:** 10.3389/fimmu.2020.01326

**Published:** 2020-06-24

**Authors:** Xiaoxiao Du, Sheng Chang, Wenzhi Guo, Shuijun Zhang, Zhonghua Klaus Chen

**Affiliations:** ^1^Henan Key Laboratory of Digestive Organ Transplantation, Open and Key Laboratory of Hepatobiliary & Pancreatic Surgery and Digestive Organ Transplantation at Henan Universities, ZhengZhou Key Laboratory of Hepatobiliary & Pancreatic Diseases and Organ Transplantation, Department of Hepatobiliary and Pancreatic Surgery, The First Affiliated Hospital of Zhengzhou University, Zhengzhou, China; ^2^Key Laboratory of Organ Transplantation, Institute of Organ Transplantation, Tongji Hospital, Tongji Medical College, Huazhong University of Science and Technology, Ministry of Education, NHC Key Laboratory of Organ Transplantation, Key Laboratory of Organ Transplantation, Chinese Academy of Medical Sciences, Wuhan, China

**Keywords:** tolerance, liver transplantation, operational tolerance, cell therapy, hematopoietic stem cell transplantation, regulatory T cells, regulatory dendritic cells

## Abstract

Liver transplantation is currently the most effective method for treating end-stage liver disease. However, recipients still need long-term immunosuppressive drug treatment to control allogeneic immune rejection, which may cause various complications and affect the long-term survival of the recipient. Many liver transplant researchers constantly pursue the induction of immune tolerance in liver transplant recipients, immunosuppression withdrawal, and the maintenance of good and stable graft function. Although allogeneic liver transplantation is more tolerated than transplantation of other solid organs, and it shows a certain incidence of spontaneous tolerance, there is still great risk for general recipients. With the gradual progress in our understanding of immune regulatory mechanisms, a variety of immune regulatory cells have been discovered, and good results have been obtained in rodent and non-human primate transplant models. As immune cell therapies can induce long-term stable tolerance, they provide a good prospect for the induction of tolerance in clinical liver transplantation. At present, many transplant centers have carried out tolerance-inducing clinical trials in liver transplant recipients, and some have achieved gratifying results. This article will review the current status of liver transplant tolerance and the research progress of different cellular immunotherapies to induce this tolerance, which can provide more support for future clinical applications.

## Introduction

Liver transplantation has been a preferred option for patients with end-stage liver disease. Since Dr. Starzl performed the first human liver transplantation in 1963, the short-term survival rate of liver transplant recipients has improved significantly, which can be attributed to advances in surgical techniques and immunosuppression (IS) agents (1). However, owing to the long-term use of IS agents, complications concerning the cardiovascular and cerebrovascular systems, diabetes, chronic renal insufficiency, infection, or tumors seriously affect the long-term survival of liver transplant recipients (2, 3).

Immunological self-tolerance refers to the ability of a healthy immune system to produce a protective immune response to pathogens and foreign antigens while maintaining tolerance to its own tissues. Therefore, transplant tolerance refers to accepting organs without the need for long-term IS and maintaining protective immunity (4). Tolerance is usually classified as complete immune tolerance, operational tolerance (OT), and prope tolerance, referred to a condition with a low, non-toxic dose of IS (5). In clinical applications, the focus of the study is OT, that is, long-term functional graft survival in a patient not requiring maintenance IS (6).

The liver is an immunologically privileged organ compared to other transplant organs such as the heart, kidney, or pancreas (7). Liver transplant recipients generally maintain a low level of IS and the hepatic graft can provide immunological protection when transplanted in combination with other solid organs (8). Since Calne first recognized the liver tolerance effect (8) in 1969, people have been enthusiastic about the induction of liver immune tolerance.

As the body's largest digestive organ, the liver has two sets of blood supplies, the portal vein and the hepatic artery. It is constantly exposed to enterically-derived blood-borne pathogens, which gives the liver a unique form of immune privilege (9). Numerous sinuses constitute the largest reticuloendothelial system in the human body and contain the largest number of specialized and non-specialized antigen-presenting cells (APC) and cells that maintain liver immune tolerance, including resident macrophages (also known as Kupffer cells), dendritic cells, hepatocytes, hepatic sinusoidal endothelial cells (LSECs), and hepatic stellate cells (HSCs) (10). When pathogen-derived products such as lipopolysaccharides pass through the liver, like an immune filter, their concentration could be reduced 100-fold, enabling the hepatic immune microenvironment to have sufficient capacity to regulate the nature and intensity of its response (11). Numerous immune regulatory mechanisms in the liver, including downregulation of co-stimulatory molecules, secretion of inhibitory cytokines, inhibition of effector T cell activation, and induction of regulatory T cells, predispose the immune response of the liver to tolerance rather than activation (12).

## Current State in Spontaneous Tolerance

Factually, in liver transplantation, spontaneous tolerance initially came from the casual clinical observation. Owing to poor compliance, infection complications, posttransplant lymphoproliferative disease (PTLD) or doctor's advice, some liver transplant recipients developed spontaneous tolerance after discontinuing IS, which has aroused great interest of transplant researchers. In 1993, Reyes et al. in Pittsburgh reported that 8 liver transplant recipients with poor compliance ceased IS from 0.5 to 11 years after transplantation, but unexpectedly developed OT. Among them, after weaving from IS, 7 recipients maintained good allograft function for 1 to 14.3 years. The remaining recipient underwent liver retransplantation after 7.7 years of IS withdrawal for viral hepatitis. In addition, 6 recipients were shown to have systemic chimerism (13).

Over nearly three decades, many liver transplant centers have conducted clinical trials of IS withdrawal in both adult and pediatric liver transplant recipients. It has been reported that normal liver function was successfully maintained in adult and child liver transplant recipients, with ~20% of patients (6–63%) achieving complete immunosuppressive withdrawal (Tables 1, 2) (14–33).

**Table 1:** Immunosuppression withdrawal trials (≥10 recipients, single center).

**ID**	**Year**	**Author**	**Transplant center**	**Adult or pediatric**	**No. of recipients**	**Donor (DD or LD)**	**HCV+ or AIH ?**	**IS**	**Time from LT to IS withdraw (Years)**	**Follow-up after IS withdraw (Months)**	**OT N (%)**	**Rejecton N (%)**	**References**
/	1997	Mazariegos	Pittsburgh	Both	95	DD	Yes	CsA/AZA/Pred	8.4 ± 4.7	35.5 (10.1–57.2)	18 (19%)	18 (19%)	(14)
/	1998 2005	Devlin; Girlanda	London	Adult	18	DD	Yes	CsA/AZA/Pred	7 (5~11)	120	2 (11%)	7 (39%)	(15, 16)
/	2001	Takatsuki	Kyoto University	Pediatric	26	LD	No	TAC	>2	25 (3~69)	6(23%)	16 (25.4%)	(17)
/	2002	Oike	Kyoto University	Pediatric	115	LD	No	TAC	/	4~96	49(42%)	20 (17%)	(18)
/	2005	Eason	New Orleans	Adult	18		Yes	TAC	>0.5	6~12	1 (6%)	11 (61%)	(19)
/	2005 2010	Tryphonopoulos	Miami	Adult	104	DD	Yes	TAC/CsA/SRL	4.1 ± 0.3	7.27 ± 0.28	23(22%)	71 (68%)	(20, 21)
/	2006 2008	Tisone; Orlando	University of Rome	Adult	34	DD	only HCV+	CsA	5.3 ± 1.7	63.5 ± 20.1	7 (20%)	26 (76.5%)	(22, 23)
/	2007	Assy	Western Ontario	Adult	26	DD	Yes	CsA/AZA	4.6 ± 1.8	12	2(8%)	15 (58%)	(24)
/	2009	Pons	Murcia	Adult	20	DD	No	CsA	40.8 ± 26.4	47.5 (10–131)	8(40%)	6 (30%)	(25)
/	2013	de la Garza	Pamplona	Adult	24	DD	No	TAC/CsA/SRL	9.3 (6~13.3)	14(8.5~22.5)	15 (63%)	2 (8.3%)	(28)
2011-02-003IA	2015	Lin	Taipei	Pediatric	16	Both	Yes	TAC	7.8 ± 5.4	40.75 ± 5.98	5 (31%)	6 (38%)	(29)
NCT02062944	2019	Levitsky	Transplant Center	Adult	15	Both	No	SRL	8.1 (4.5~12)	18 (12~24)Months	8 (53%)	6 (40%)	(32)

*CNI, calcineurin inhibitor; CsA, Cyclosporine A; Pred, prednisone; SRL, sirolimus; DD, deceased donor; LD, living donor; LT, liver transplant; IS, immunosuppression; OT, operational tolerance*.

**Table 2:** Immunosuppression withdrawal trials (≥10 recipients, Mul-Center).

**ID**	**Year**	**Author**	**Country**	**Adult or pediatric**	**No. of recipients**	**Donor (DD or LD)**	**HCV+ or AIH ?**	**IS**	**Time from LT to IS withdraw (Years)**	**Follow-up after IS withdraw (Months)**	**OT N (%)**	**Rejection N (%)**	**References**
NCT00647283	2012	Benitez	Spain/UK/Italy	Adults	98	Not mention	No AIH, HCV+ included	TAC/CsA/AZA	8.6 ± 3.9	48.9 ± 7.7	41 (42%)	57 (58%)	(27)
WISP-R, NCT00320606	2012 2017	Feng S	U.S.	Pediatric	20	LD	No	TAC/CsA	8.5 (6.4 ~ 10.75)	48(1 quit at 33.3 Months)	12(60%)	2(10%); 5(25%)not certain rejection	(26, 30)
ITN030ST	2019	Jucaud	U.S.	Adults	31	Not mention	No AIH, HCV+ included	Single CNI	1	14(12.3~24.3)	9(29%)	13(42%)	(31)
ITN030ST A-WISH NCT00135694	2019	Shaked	U.S.	Adults	77	DD	No AIH, HCV+ included	single CNI or antimetabolite	1~2	24	10(18%)	32(42%)	(33)

*CNI, calcineurin inhibitor; CsA, Cyclosporine A; Pred, prednisone; SRL, sirolimus; DD, deceased donor; LD, living donor; LT, liver transplant; IS, immunosuppression; OT, operational tolerance*.

Sanchez-Fueyo et al. of the Hospital Clinic of Barcelona conducted a prospective and multicenter IS withdrawal clinical trial in 98 liver transplant recipients (NCT00647283). Of these, 41 achieved clinical tolerance (41.8%) and 57 developed mild rejection (58.2%), which was followed by remission within 5.6 months. During the 3-year follow-up after IS withdrawal, no significant histological damage was found in liver biopsies of the tolerant recipients. Statistical analysis showed that years post-transplantation correlated positively with tolerance induction and could be the strongest predictor (27).

Comparing with calcineurin inhibitors (CNI), sirolimus, a kind of rapamycin inhibitor (mTOR-I), has no significant drug toxicity, such as nephrotoxicity, hypertension, diabetes, infections and neoplasms (34). Another advantage of sirolimus is its immunomodulatory ability that could facilitate safe IS withdrawal (35). To assess if sirolimus could increase the tolerability in liver transplant recipients, Levitsky et al., at Northwestern University, performed a prospective clinical trial of single sirolimus withdrawal (NCT02062944). They recruited 15 recipients with non-viral or immunological hepatitis more than 3 years after liver transplantation. After 12 months from sirolimus withdraw, it showed that 8 recipients (53%) achieved OT. Of the other 7 patients, 3 failed IS withdrawal, 3 developed moderate cellular rejection (TCMR) on liver biopsies at the end of the study, and 1 was withdrawn from the trial owing to adrenal metastasis of hepatocellular carcinoma (32). The current result showed the OT rate of sirolimus was comparable to CNI-withdrawal studies. Except for free CNI toxicity, whether liver recipients can benefit more from sirolimus withdrawal, it still needs more and larger trials.

For pediatric liver transplant recipients, Feng et al. in the University of California conducted a multicenter, prospective clinical trial of IS withdrawal (WISP-R, NCT00320606). A total of 12 (60%) of the 20 enrolled pediatric recipients achieved clinical tolerance, and during follow-up for more than 2 years after IS withdrawal, the graft function was normal and there was no significant change in biopsy compared with baseline. A total of 3 recipients underwent acute rejection (*n* = 2) or uncertain rejection (*n* = 1) during IS withdrawal, and 4 recipients failed to achieve clinical tolerance owing to uncertain acute rejection within 1 year of drug withdrawal. Their graft function recovered to normal after increased or restarted IS. Another recipient was withdrawn from the study after IS withdrawal for violating exclusion criteria. Similar to the results of the adult study, the time after transplantation was significantly longer in the tolerance group than in the non-tolerance group, suggesting that the time after transplantation is an important predictor of tolerance formation (26). Of 12 OT recipients followed for 5 years, 9 cases were positive for class I or class II DSA, but no cases resulted in chronic rejection, graft loss, or death. According to the graft biopsy, there was no progressive increase in inflammation or fibrosis, suggesting that liver grafts after immune retreat can maintain stability during a certain period of time (30).

There are also many studies focused on biomarkers that can predict immune tolerance. Bohne, et al. found that recipients with spontaneous tolerance show an increased number of natural killer (NK) cells and γδT cells in peripheral blood. High levels of hepcidin in liver tissues and ferritin in the serum, increased iron deposits in hepatocytes, and high expression of the related liver tissue genes can accurately predict the outcome for a group of independent patients with IS withdrawal (36). Mazariegos et al. showed that the ratio of monocytoid dendritic cells (mDC) and plasmacytoid dendritic cells (pDC) precursors in the peripheral blood of patients with tolerance increased significantly compared to the healthy control group and the IS maintenance group (37). Levitsky et al. also found that, compared with the baseline, the tolerogenic dendritic cells (tolDC), regulatory B cells (Breg), and cell phenotypes associated with chronic antigen presentation in peripheral blood of the OT group was significantly higher than that of the non-OT group. In addition, gene signatures in peripheral blood/biopsy tissue showed that 12/14 LTR could accurately predict tolerance (32). Chruscinski et al. performed a clinical trial (NCT02541916) for the predictive value of gene signatures in peripheral blood/biopsy tissue. Preliminary results suggest that 5 of the 9 patients, consistent with the inclusion criteria, had discontinued IS for more than 2 years (38). However, the feasibility of this method still needs to be verified by adequate prospective, multicenter, large-scale follow-up trials.

Long-term studies on the safety of immunosuppressive IS withdrawal regimens are inconclusive, and most of them lack evidence of invasive liver biopsy. However, direct comparisons of these trials are difficult because of the lack of standardization. According to the current research results, the acute rejection rate after IS withdrawal varies from 12 to 76% (Tables 1, 2), but it is generally moderate and almost reversible. Chronic rejection is rare (0–6%), and the incidence of graft loss owing to rejection is extremely low (39, 40). Over time, however, the prevalence and severity of chronic graft injury such as subclinical rejection, chronic portal inflammation, borderline hepatitis, and/or fibrosis (periportal and/or perivenous) would increase (41–51). Ten years after transplantation, most studies report that normal histology is present in up to 30% of allografts; bridging fibrosis and/or cirrhosis may be equally common, accounting for about 60% (42, 45, 52). The transcriptome analysis of liver tissue revealed an expression profile very similar to that of T-cell mediated rejection (53). Notably, more than 90 percent of patients who stopped IS 20 years after the transplant did not experience rejection (27). To date, there is no definitive data suggesting that progressively abnormal histology leads to loss of liver graft or death of recipient. However, the OT is not a permanent stable state, still needed regular inspection and to deal with in time.

Because of the difficulty to conduct prospective, multicenter, and longitudinal long-term studies on clinical transplant tolerance, the risk of IS withdrawal in liver transplant recipients remains uncertain. However, based upon a broad understanding of liver disease, increased fibrosis in the allograft indicates the development of portal hypertension and associated complications. IS withdrawal alone could make recipients at the risk of re-transplantation after some years, especially for pediatric recipients. Therefore, there is a wide area of research for the development of induced liver tolerance, especially with cell therapies.

## Hematopoietic Stem Cell Transplantation (HSCT) for the Induction of Liver Transplantation Tolerance

Mixed hematopoietic chimerism is associated with alloantigen tolerance, a phenomenon first identified by Owen in freemartin cattle (fraternal twins sharing placental circulation retain allogeneic tolerance to each other after birth) in 1945 (54). Subsequently, Kashiwagi and Starzl discovered donor immunoglobulin in the peripheral blood of liver recipients in 1969, proposing the concept of chimerism in human transplantation (55).

In a series of pioneering studies that began in 1961, Till and McCulloch demonstrated that bone marrow consists of a group of cells, known as hematopoietic stem cells (HSCs), which have the ability to self-renew and differentiate into multiple myeloid cell types (56–58). Afterward, Starzl et al. observed persistent multilineage hematopoietic microchimera (defined as <1% donor cells) in both lymphoid and non-lymphoid tissues of long-lived liver or kidney transplant recipients, including patients on IS for many years after transplantation in 1993 (59). Over the past 30 years, many researchers have been exploring ways to induce tolerance of solid organ transplantation (SOT) using HSCT. Both autologous and allogeneic HSCT have been applied to induce transplant tolerance clinically (60–63).

Clinically, hematopoietic stem cells were first harvested from the bone marrow of the ilium (64). In the 1990s, the protocol to mobilize stem cells into peripheral circulation with granulocyte colony-stimulating factor (G-CSF) and isolate from PBMC by magnetic activated cell sorting (MACS) or flow sorting, thereby separating CD34+ cells, greatly amplified the clinical application of HSCT. Subsequent studies show that combination with chemokine receptor 4 antagonist (AMD3100) and G-CSF can mobilize stem cells more effectively (65).

### Allogeneic HSCT Inducing Chimerism

After lymphoablation of the recipient, transfusion of allogeneic donor bone marrow can lead to mixed hematopoietic chimerism, where genetically different donor HSCs are implanted into the host and differentiate into donor-derived lymphocytes that coexist with the host. Central tolerance is a key mechanism of allograft tolerance induced by long-term HSCs (66). When donor bone marrow is injected into the host that has undergone lymphoablation, such as total lymphoid irradiation (TLI) and anti-thymocyte globulin (ATG), HSCs are implanted into the recipient bone marrow and thymus for differentiation, and the host immune system is repopulated with various lymphocyte cells from the donor following by the immunoreconstitution. The presence of donor progenitor cells in the thymus leads to the apoptosis of T cells that recognize the donor antigen expressed by the transplanted organ itself without developing and donor-reactive T cells would undergo clonal deletion, so the host can tolerate the allograft (67). The coexistence of host and donor hematopoietic cells is called chimera, and it is this chimera state in the host that drives central tolerance. According to the percentage of hematopoietic cells of donor origin, the chimerism was divided into microchimerism (donor <1%) and two forms of macrochimerism: full chimerism (donor ~100%) and mixed chimerism (donor >1% but <100%) (68). Although central tolerance is the primary mechanism for HSCs-induced tolerance to homo-antigens, it may be incomplete, in part because not all donor antigens are expressed by HSCs in the host thymus. Furthermore, T lymphocytes with low affinity for their own antigens may escape the selection process, thereby entering the peripheral lymphocyte cycle. In fact, peripheral mechanisms are needed to maintain immune tolerance when the self-reactive T cell subsets evade the thymus selection process. In the transplant environment, a mild pre-treatment regimen designed to induce chimerism can also control the survival of mature alloreactive T cells through peripheral regulation mechanisms, resulting in clone deletion, anergy, or apoptosis of the extra-thymic alloreactive T lymphocytes (66, 67, 69–71). Persistent microchimerism may also be an important determinant for long-term graft survival and transplant tolerance (72, 73).

Since the mid-twentieth century, scientists have used HSC transfusion by injecting donor bone marrow to alter host immune responses in a variety of autoimmunity diseases and solid organ transplantation (SOT) (74, 75). The ideal state of clinical transplant tolerance is to combine HSCT with SOT from the same donor to form a stable hematopoietic chimera of donor and recipient (76). However, myeloablative therapy carries significant risks, most notably graft vs. host disease (GVHD) and severe infections, which are too high-risk to be applied in liver transplants routinely. Therefore, researchers investigated protocols for non-myeloablative bone marrow transplant, including co-stimulatory molecules blockade, low dose irradiation, T cell depletion by monoclonal antibody, etc. (77–79). Chimerism has always been the main method of inducing tolerance in renal transplantation. Clinical studies on transplant tolerance induced by bone marrow chimerism in renal allografts have also achieved gratifying results. In 2008, Kawai et al. reported the first successful application of mixed chimerism tolerance in human kidney transplantation without long-term maintenance of IS (80). The authors then reported that 5 out of 10 kidney recipients had achieved transplant tolerance. Although the detectable duration of chimerism was transient, it was observed that donor-specific mixed lymphocyte response (MLR) and CTL activity decreased *in vitro* and FOXP3 mRNA level increased *in vivo* (81). Based on the enriching of HSC with tolerogenic CD8+/TCR− facilitating cells (FC) and the depleting of GVHD-producing cells, Leventhal et al. performed a clinical trial in 19 patients with uremia undergoing combined HLA-mismatched hematopoietic stem cell/kidney transplantation. Then 12 of them achieved stable chimerism and OT status of 8–48 months without GVHD after IS withdraw (82). Scandling et al. reported that 16 of 22 HLA-matched patients who had received the same treatment regimen established a persistent mixed chimera (>12 months), with successful withdrawal from immunosuppressants. Renal graft function was stable for up to 7 years after withdrawal, and no incidence of GVHD or rejection was observed (83).

In the field of liver transplantation, there have been some case reports and clinical trials with HSCT transfusion. Ringden et al. reported a liver cancer patient who received HSCT from the same donor after liver transplantation, underwent preoperative myeloablation, achieved chimerism, but subsequently died of opportunistic infection (84). Donckier et al. recruited 5 patients with advanced hepatocellular carcinoma (HCC) who underwent living liver transplantation and received donor CD34+ stem cell transfusion based on the induction regimen of non-myeloablative therapy. Two patients successfully stopped IS without allograft rejection. Three patients developed acute cellular rejection after immunosuppressant withdrawal, two of which were given steroid pulse therapy, whereas the other was reintroduced to calcineurin inhibitor (CNI) immunosuppressive therapy, with no observation of macrochimerism (85, 86). Tryphonopoulos et al. recruited 45 adults who received cadaver livers and subsequently underwent transfusion of donor bone marrow cells on the day of the transplant. IS were discontinued for more than 3 years, starting 3 years after surgery. Acute rejection occurred in 69% of the treated patients, and immunosuppressive therapy was successfully withdrawn in 22.2% of the treated recipients. However, there was no significant increase in the success of withdrawal and chimerism levels, compared to patients who did not receive bone marrow transplants (20). Liver transplantation is mainly performed from cadaver donors, and recipients generally suffer from severe diseases during the perioperative period, as well as postoperative coagulation and circulatory dysfunction, which may lead to serious infection and tumor recurrence.

Surprisingly, Alexander et al. reported a successful case that a 9-year-old girl with type O, RhD negative underwent RhD blood type conversion to positive after receiving the liver graft of a male donor with type O, RhD positive. Furthermore, CD19 + B cells (XY) were found in the sample of bone marrow puncture. Peripheral blood lymphocyte analysis showed that 94% of T cells came from male and 6% from female; 98% of the B cells came from male and 2% from female; 100% of the granulocytes and NK cells came from male. These results support the formation of chimera. When IS was withdrawn 14 months after transplantation, both the graft and the recipient were healthy for 5 years without GVHD or acute rejection. This indicates that fully tolerated chimera can still occur under certain conditions (87).

For the application of allogeneic HSCT in liver transplant tolerance and chimerism, there are some challenges to overcome, e.g., the risk of serious infection, coagulation and circulatory dysfunction following the myeloablative, the tumor recurrence and the permanence of existence in recipient.

### Autologous HSCT Inducing Chimerism

Autologous HSCT is performed by pre-collecting and cryo-preserving autologous bone marrow or peripheral blood stem cells isolated from the patient and then re-transfusing after myeloablative treatment to reconstruct the immune system, which could lead to a more tolerant immune system (88, 89). In autologous stem cell transplantation, the cells come from the recipient, which theoretically prevents the possibility of immune rejection or GVHD. Moreover, autologous bone marrow or peripheral stem cells are easier to obtain and store.

Although there are some differences between allogeneic and autologous HSCT with regard to tolerance mechanisms, they both attempt to reconstruct the recipient immune system and achieve immune tolerance through “re-education.” The basic principle of autologous HSCT is to first eliminate reactive and memory immunity and then regenerate the immune system; that is, to exhaust autoreactive and memory T and B cells through a myeloablative or non-myeloablative regimen, followed by reconstruction of immune tolerance (89, 90). The immune monitoring analyses have shown that this can recreate new auto-tolerant immune T and B cell banks, enhance immune regulation mechanisms, and induce changes in the recipient's anti-inflammatory environment (63, 91–95). Muraro et al. found a large number of new T cell clones emerged after autologous HSCT in patients with multiple sclerosis (MS), substituting for the original T cell receptor (TCR) bank and showing a greater diversity of TCR spectrum (63). The immune cell gene expression profiles showed that the number of CD3 + cells remained low after autologous HSCT, and the number of CD8 + cells could return to normal after 3 months postoperatively (92). Another important phenotypic observation is that the recipient's CD4 + CD25 + FoxP3 + regulatory T cells (Tregs) were significantly increased (96–98) and so were the CD8 + Foxp3 + Tregs (99), compared to the preoperative state. The ratio of Bregs increased briefly after autologous HSCT and remained at a higher level for at least 2 years after transplantation, suggesting that Bregs may be involved in the reconstruction of self-tolerance after AHSCT (95, 100). In addition, a variety of cells, such as IL-2, IL-4, IL-6, IL-8, IL-10, IL-17, IL-18, IFN-γ, TNF-α, and TGF-β play an essential role in immune reconstruction and regulation (95, 97).

In recent years, autologous HSCT has been used in clinical trials to eliminate various types of refractory autoimmune diseases such as multiple sclerosis, systemic sclerosis (SSc), systemic lupus erythematous (SLE), rheumatoid arthritis (RA), Crohn's disease, juvenile arthritis, and type 1 diabetes (T1D), presenting better results than traditional therapies and showing promising prospects for long-term remission of autoimmune diseases without IS (90, 93, 95, 101–105). At present, autologous HSCT has also been widely used in various types of liver cirrhosis, improving liver function in varying degrees (106–109). Coupled with the study of transplant animal models, these studies provide abundant theoretical basis for expanding autologous HSCT application in organ transplantation (110–112).

In Toronto University (Canada), Levy et al. have performed a clinical trial on autologous HSCT for allogeneic organ transplant tolerance (ASCOTT) (NCT02549586) (38). Six liver transplant recipients were recruited, five of whom were enrolled. The HSCs of patients were mobilized, purified, and cryopreserved ahead of liver transplant. After immune ablation (Busulfan + Cyclophosphamide + rabbit anti-thymocyte globulin), the patients received autologous HSCT and liver transplantation. IS was withdrawn in five patients with evidence of deletion of alloreactive T cell clones. Two of them were still healthy at 406 and 518 days after HSCT; one died of heart failure at 212 days, one patient received re-transplantation at 166 days after HSCT owing to complications of venous occlusive disease, and one patient died of erythrocytic syndrome at 87 days after HSCT. However, non-hematological toxicity in grades 3–4 was found in almost all patients. It is suggested that there is a certain application prospect of HSCT-induced immune tolerance after liver transplantation, but the potential toxicity is an important problem to be solved.

## CD4 + Regulatory T Cells in Liver Transplant Tolerance

Under physiological conditions, Tregs account for 5–10% of CD4 + T cells in peripheral blood. Tregs are characterized by high stable expression of CD25 and FoxP3, and are divided into natural regulatory T cells (nTreg) produced in the thymus and peripherally induced regulatory T cells (iTreg) (113). Modern research shows that CD4 + Treg is the key to control self-tolerance. The combination can induce peripheral tolerance to autoantigens and alloantigens through a variety of mechanisms, mainly cell-to-cell contact-induced cell lysis, local depletion of IL-2, inhibition of DC maturity, downregulation of DC function, secretion of immunosuppressive cytokines (such as IL-10, IL-35, and TGF-β), etc. (114). In addition, Tregs can migrate to the inflammation site, and their inhibitory activity is usually located in the inflammation site, without significant effects on overall immunity (115).

In rodent models of liver transplantation tolerance, Tregs are present in increased proportion in liver grafts and are involved in the induction of liver tolerance (116, 117). Relevant clinical studies have also shown that, in OT liver transplantation recipients, the proportion of Tregs in peripheral blood and liver increases, which shows a protective effect on liver allografts (118, 119). Therefore, the use of Tregs to mediate transplant tolerance is an important part of transplant immunology research.

Treg adoptive infusion is a method to induce tolerance, where the principle is to tilt the immune response toward Treg dominance, rather than to cause rejection of T effector cells, in order to reduce the dependence of patients receiving solid organ transplantation on immunosuppressant drugs.

It is now generally accepted that the best method to make Treg clinically is to effectively expand it *in vitro* and maintain high purity and inhibitory activity (115). Under certain culture conditions, human Treg can be expanded to 100–1,000 times in 2 weeks (120, 121). The required cell dose varies depending on the type of disease and the presence or absence of combination therapy (115, 122, 123). Treg separation and purification methods mainly include MACS and flow cytometry sorting.

Some researchers have used the chimeric antigen receptor (CAR) technology to produce donor antigen-specific Treg (CAR-Treg) and can overcome the limitation of alloantigen-stimulation-based protocols *in vitro*. In these studies, CARs could be developed to redirect Tregs toward a specific donor leucocyte antigen (HLA) class I molecule (HLA-A2). Unlike HLA class II, the selected donor HLA class I is expressed ubiquitously in grafts. Compared with polyclonal Tregs, CAR-Treg could have better safety, stability, and effectiveness in theory and have strong therapeutic potential to protect allograft (124–127).

Clinical trials of various autoimmune diseases and GVHD have confirmed the safety and feasibility of adoptive Treg infusions (128–131). Clinical reports of adoptive infusion of Treg in kidney transplant recipients have demonstrated the safety of this method in solid organ transplantation, and, through the method of isotope plutonium labeling (^3^H), it has been found that Tregs can still be detectable for up to 1 year after infusion (132, 133).

Currently, multiple transplant centers around the world are conducting clinical trials of liver transplantation Treg treatment. Todo et al. from the Hokkaido University in Japan, studied 10 liver transplant recipients who received a single dose of donor antigen-specific Treg. Tregs from recipient lymphocytes were amplified by co-culture with irradiated donor cells in the presence of anti-CD80/86 monoclonal antibodies *in vitro* for 2 weeks. CD4 + CD25 + FoxP3 + Tregs were amplified 3–6-fold to 28.1% of CD4 + cells and still maintained inhibitory activity. On the 13th day after the operation, the cells were infused back to the recipient with 0.23~6.37 × 10^6^ cell/Kg intravenously. IS was gradually reduced during 6 months and withdrawn 18 months postoperatively. These recipients were subjected to rigorous monitoring, including liver biopsies, T cell activity assessments, and level of donor-specific antibodies. After 16 to 33 months of follow-up, 7 patients achieved OT without rejection and 4 remained IS-free for 24 months. Mild rejection occurred in 3 patients, and low dose IS was maintained afterward (121).

Safinia et al., at London University (UK), performed a combined I/IIa clinical trial ThRIL (NCT02166177) for the application of Treg immunotherapy in the field of liver transplantation. Tregs were isolated from liver transplant recipients by Good manufacturing practice (GMP) separation technology based on CliniMACS sorting. IL-2 and rapamycin were used for Tregs expansion *in vitro*. A stable Treg population (purity of CD4 + CD25 + FOXP3 +> 95%) can be obtained in 36 days, reaching a sufficient number for its clinical application (120). With stimulation by rapamycin, the amplified Tregs could maintain high levels of FOXP3, CD127lo, and CTLA4, and, with continued expression of CD62L and CXCR3, ensure the stability and functionality of Treg amplification, which could prevent Treg from transforming into Th17 cells in the presence of pro-inflammatory cytokines (134). Nine liver transplant recipients have received autologous polyclonal Treg infusions, showing that the procedure is safe, does not increase the incidence of infection or cancer, and can temporarily increase circulating Treg pools and reduce anti-donor T cell reaction (135).

The current research has opened the door for adoptive infusion of Treg in liver transplantation induction therapy, showing good application prospects. Subsequent research may need to focus on isolation purity, functional induction, CAR-Treg, and clinical induction protocols for OT of Treg *in vitro*.

## Regulatory Dendritic Cells in Liver Transplant Tolerance

In 1973, Steinman et al. found a cell type with a “star shape,” or dendritic morphology, found in the preparation of adherent spleen cells and named dendritic cell (DC) (136). It has been recognized that DCs are a group of highly heterogeneous cell populations derived from the myeloid or lymphoid, which are widely distributed in all tissues and organs and are the most powerful APC in the body, regulating both innate and adaptive immunity and playing an important role in promoting self-tolerance in healthy homeostasis (137, 138). In humans, according to their cell morphology and function, DCs are divided into two mean lineages of CD11c + conventional DCs (cDCs) (HLA-DR + CD11c +) and CD11c- plasmocyte-like DCs (pDCs) (HLA-DR + CD123 +) (139).

In 1996, Steptoe and Thomson defined the DC population that can induce immune tolerance *in vivo* as tolerogenic DC (tolDC) (140). However, it is still unclear whether tolDCs constitute a particular lineage or just reflect a specific activation state of DCs (141). In 2003, Sato et al. named the tolerogenic DCs they cultured *in vitro* as "regulatory DCs (regDC),” because they had the ability to inhibit T cell activation, induce T cell anergy, and induce Tregs. They could also maintain strong immunoregulatory properties in inflammatory conditions and have the potential to resist multiple immune diseases (142, 143). This nomenclature has also been widely used in classic tolerogenic DCs and their derivatives (144–148). Currently, two methods of naming such tolerant DCs are both widely used. Over the past 20 years, a large number of studies have found that regDCs could be used to treat various autoimmune diseases in animal models, such as T1D, SSc, RA, Cohn's Disease, etc. (149–152), and to induce tolerance of in GVHD and allografts (115, 143, 153, 154). They also have good clinical application prospects in the field of liver transplant tolerance (139, 155).

The phenotypic characteristics of regDC include low expression of MHC class I and II molecules and T cell co-stimulatory molecules (CD80/B7.1, CD86/B7.2, CD40, OX40L), T cell co-inhibition of ligands (such as programmed death Ligand 1 PD-L1), high expression of death-inducing ligands (FasL), and low expression of adhesion molecules (156, 157). Unlike immature DCs, there are indications that tolerability of regDCs is the result of a specific transcription program, rather than the preservation of immature status (158).

RegDCs retain the ability to present antigens to specific T cells, and they can also build up peripheral tolerance through different immunoregulatory mechanisms. These related promotion mechanisms include the following:

- T cell anergy and T cell clonal deletion (159, 160);
- Apoptosis in naive and memory T cells through increased expression of Fas (CD95)/FasL and indoleamine 2,3-dioxygenase (IDO) (161, 162);
- Inducing and expanding regulatory lymphocytes, including Tregs (163, 164) and Bregs (165);
- Producing double negative (CD3 [+] CD4 [−] CD8 [−]) T cells (166);
- Development of tolerance by increasing the expression and release of immune regulatory molecules, such as the anti-inflammatory cytokines IL-10, TGF-b, NO, and HO-1 (167–170), the apoptosis-inducing PD-L 1, PD-L2, and human leukocytes Ag-G (HLA-G), and the tumor necrosis factor (TNF) (161, 164, 171, 172).

Recent studies have shown that exosomes released by regDCs are also involved in the induction and maintenance of peripheral T cell tolerance (172–174).

Although DCs are widely distributed in tissues, their proportion is very low. Immature DCs (imDCs) are tolerogenic in the body, but they are also unstable and may differentiate into immunogenic DCs in inflammatory conditions. Therefore, it is very important to establish a mature system for regDC culture *in vitro* to obtain a sufficient number of functional and stable regDCs.

DCs in the immune system act as “immune checkpoints,” with the key role of turning immune signals on or off. A large number of anti-inflammatory and immunosuppressive mediators can promote tolerogenic phenotypes by interfering with DC differentiation or activating checkpoints (157). Researchers have explored different strategies for generating stable regDCs, some of which have been performed in clinical trials, but a consensus hasn't been reached on the best approach yet (115, 157, 172, 175, 176).

Currently, regDCs *in vitro* are mainly derived from rodent bone marrow cells and human peripheral blood mononuclear cells, as it is easier and less invasive than to operate in humans, and abundant DC precursors are also available. Granulocyte-macrophage colony-stimulating factor (GM-CSF) ± IL-4 can be added to fresh or frozen blood mononuclear cells or their precursors to promote the differentiation of myeloid tolDCs (143, 175). Then, one or more anti-inflammatory and immunosuppressive agents should also be added to inhibit their maturation and promote tolerance. These agents include anti-inflammatory cytokines (such as IL-10, TGF-β, TNF-α), anti-inflammatory/IS drugs (CNI, rapamycin, mycophenolate, corticosteroids, or aspirin), Vitamin D3, Prostaglandin E2, retinoids, and HLA-G, tissue factors, such as hepatocyte growth factor (HGF) and vasoactive intestinal peptide (VIP), etc. (139, 143, 153, 177–180).

In rodent and non-human primate transplantation models, adoptive infusion of DCreg prior to transplantation can prolong the survival of allografts and promote specific tolerance to the graft either alone, or in combination with short-term IS (144, 154, 181–183). Clinical trials on the safety and effectiveness of regDC have been conducted for a variety of autoimmune diseases, including T1D, RA, multiple sclerosis (MS), Crohn's Disease (184–188) (NCT02618902, NCT02903537, NCT01352858, NCT00445913), and renal transplant rejection (137) (NCT 0364265, NCT02252055). So far, although there are no long-term results, it has been confirmed that a regDC regimen is safe and feasible without significant side effects, and that the patient's compliance is good. These results provide a good theoretical guide for a regDC therapeutic schedule for liver transplantation.

In Pittsburgh University (USA), Thomson et al. performed a single-center, phase I/II clinical trial on regDC in living donor liver transplantation (NCT03164265). The study recruited low-risk living donor liver transplantation (LDLT) recipients, isolated monocytes from peripheral blood from potential living organ donors, and cultured cells with GM-CSF, IL-4, VitD3, and IL-10 for 7 days *in vitro*. GMP-grade donor-derived regDCs could be induced (153, 189), and a single intravenous infusion of 2.5–10 × 10 ^6^ donor-derived regDCs/kg was administered 7 days before the surgery. Meanwhile, half a dose of mycophenolic acid (MPA) was given, without ATG or Ab. MPA and Tac were administrated within 6 months after transplantation. At 6 months after transplantation, recipients who meet specific criteria [non-rejection and liver function allowance tests (LFTs)] may gradually discontinue MPA. TAC withdrawal assessments are performed 1 year after transplantation and then discontinued gradually to achieve complete IS withdrawal 18 months after liver transplantation. The recipients will be followed up for 3 years from IS withdrawal. During the follow-up period, clinical data and peripheral blood were regularly collected and analyzed to evaluate changes in liver function, renal function, donor-specific antigen (DSA) levels, cardiovascular risk factors, and quality of life. Meanwhile, liver biopsy is to be performed after 1 and 3 years from the withdrawal of IS (146). This study is still in the research stage, but we are looking forward to the results.

Autologous DCs seem to be more feasible than donor-derived DCs, especially for liver transplantation from deceased donors, as it can avoid the risk of sensitization. Autologous regDC infusion with or without donor antigen pulse has shown good tolerogenic effects in animal transplant model studies (179, 190). Some studies have shown that autologous tolDC is more effective than donor DC in delayed transplant rejection (180, 191). Under the leadership of the European Union, in Nantes University (France), Moreau A. et al. conducted a Phase I/II (feasibility/safety) “one study” (www. onestudy.org) on kidney transplant recipients with autologous tolDCs infusion (NCT0225055) (192), and its clinical effects are still being observed.

There are few clinical trials about regDC in the induction of liver transplantation tolerance, and still in the observation stage up to now. As a kind of powerful immune-regulating cell, the prospect of regDC is still thrilling in liver transplantation tolerance. For the successful conversion from preclinical researches to clinical application, researches still have many issues to be studied extensively, such as the further optimization of the regDC induction scheme to extend its half-life, the stability of immunomodulatory function, and the administration scheme of IS. However, the further exploration of methods to induce immune tolerance will also improve our understanding of the biological characteristics of DC and the mechanisms of tolerance.

## Summary and Future Development

Immune tolerance has always been the “holy grail” in the field of organ transplantation. On the basis of a large number of preclinical studies, various cell therapies could hold promising prospects for inducing liver tolerance. This article reviews the related research and progress regarding spontaneous tolerance and the HSCT, Tregs, and regDCs strategies in the field of liver transplant tolerance. There are many other cells not reviewed that may also have the potential to induce liver transplant tolerance, such as mesenchymal stem cells, regulatory macrophages, regulatory B cells, and bone marrow-derived immunosuppressive cells. At present, the clinical trials of various cell therapies are still in the early stages, and most of them are single-center studies. Thus, there is no clear clinical effect, the purity and stability of cell-induced therapy and its safety for long-term recipients should still be explored for a long time, as many issues need to be observed.

The immune system is extremely delicate and complex. It may be difficult to achieve immune tolerance using only one type of tolerant cell or one mechanism. It may be necessary to consider different mechanisms in combination with different immune cells or drugs. The development of immunologic surveillance and tolerance markers is also critical. This could develop personalized tolerance induction programs for transplant recipients and could guide the timing of immunosuppressive drug withdrawal or early detection of rejection, infection, or tumors.

With the in-depth development of multi-field, multi-disciplinary, and multi-level research, the application of various new experimental methods can provide more possibilities and theoretical guidance for liver transplant tolerance. With the development of multi-center clinical trials, we are optimistic about the good prospects for liver transplant tolerance.

## Author Contributions

ZC devised this topic. XD wrote the manuscript. ZC, SC, WG, and SZ helped to revise the manuscript. All authors contributed to the article and approved the submitted version.

## Conflict of Interest

The authors declare that the research was conducted in the absence of any commercial or financial relationships that could be construed as a potential conflict of interest.

## Abbreviations

IS: immunosuppression
OT: operational tolerance
APC: antigen-presenting cells
NK cells: natural killer cells
PBMC: peripheral blood mononuclear cells
HSCs: hematopoietic stem cells
HSCT: hematopoietic stem cell transplantation
GVHD: graft vs. host disease
Tregs: regulatory T cells
DC: dendritic cells
regDC: regulatory dendritic cells
tolDC: tolerogenic dendritic cells
GM-CSF: granulocyte-macrophage colony-stimulating factor
GMP: Good manufacturing practice.
